# The relationship between apolipoprotein CIII gene polymorphism and serum lipid levels in Han Chinese males^☆^

**DOI:** 10.1016/j.mgene.2013.09.005

**Published:** 2013-11-15

**Authors:** Shu Su, Min Tang, Mingjun Zhang, Chunquan Cheng, Xiaojun Tang

**Affiliations:** a School of Public Health and Management, Chongqing Medical University, Chongqing, China; b Department of Public Health Sciences, Karolinska Institutet, Stockholm, Sweden; c College of Laboratory Medicine, Chongqing Medical University, Chongqing, China; d The First People's Hospital of Jiulongpo District, Chongqing, China; e Chonggang Hospital, Chongqing, China

**Keywords:** ApoCIII, Apolipoprotein CIII, TRL, Triglyceride-rich lipoprotein, BMI, Physique index, WHR, Waist to hip ratio, IR, Insulin resistance, TC, Total cholesterol, TG, Triacylglycerol, HDL-C, High density lipoprotein-cholesterol, LDL-C, Low density lipoprotein-cholesterol, ANOVA, Analysis of variance, HTG, Hypertriglyceridaemia, ApoCIII gene, Polymorphism, Serum lipids

## Abstract

**Background:**

Although apolipoprotein (apo) CIII gene polymorphisms have proved to be related to the increased serum lipid level in foreign studies, the results have not always been consistent among diverse populations. In addition, the research focuses on the impact of the apoCIII on the serum lipid levels of Han Chinese subjects which was not conducted before.

**Objective:**

To explore the relationship between the apoCIII gene C3175G and T3206G polymorphisms and serum lipid levels as well as other risk factors for hyperlipidaemia, in Han Chinese males.

**Method:**

A total of 337 healthy male participants undergoing physical examinations were randomly selected from two hospitals in Chongqing, China. Through DNA sequencing, apoCIII gene C3175G and T3206G polymorphisms were identified and their relationships with serum lipid levels were further analysed.

**Results:**

Carriers of apoCIII^3175^ GG genotypes have higher levels of TG than other genotypes (P < 0.05). After the stratified selection of triacylglycerol (TG), G gene loci of apoCIIIT3206G are associated with decreasing the content of total cholesterol (TC) and low density lipoprotein-cholesterol (LDL-C) in relatively high TG group while the G gene loci of apoCIIIC3175G have an inverse effect. The outcome of TG unconditional logistic regression shows that the G gene loci of apoCIIIT3206G polymorphism are also beneficial for decreasing TG.

**Conclusion:**

The detection of TG in apoCIII^3175^ GG genotype carriers is an efficient predictor of hypertriglyceridaemia in Han Chinese males. The G gene loci of apoCIII^3206^ may be beneficial for decreasing serum lipid level.

## Highlights

• Our research has tried its best to avoid all possible factors associated with lipids.
• The method has been adopted in new DNA sequencing.
• No previous similar research has worked on it, and we filled the gap.
• The outcome is accurate and exciting.

## 1 Introduction

The human apolipoprotein CIII (apoCIII) gene is located in the q23 region of the long arm of the 11th chromosome in humans. The length of this gene is about 311 kb and it has 4 exons and 3 introns. ApoCIII is a water soluble, low molecular weight protein. It contains abundant triglyceride-rich lipoprotein (TRL), the catabolism of which plays an important regulatory role. It is an inhibitor of lipoprotein lipase and can also restrict the liver's absorption of TRL and its remnants. Previous studies have shown that an increase in apoCIII is an important feature recognized in patients with high blood triglycerides. ApoCIII levels in plasma are positively correlated with the concentration of triglycerides in plasma; in other words, the level of apoCIII indicates the severity of high blood triglycerides (Bobik, 2008). Meanwhile, it is also found that the apoCIII gene has many polymorphic loci. Therefore, the exploration of the apoCIII gene which can prompt early hyperlipidaemia in a population could be a significant factor in the prevention of cardiovascular disease.

In some Western countries, researchers have reported that polymorphisms of apoCIII genes C3175G and T3206G impact rising triglyceride levels (Masana et al., 2001, Souverein et al., 2005). However, different races carry different genes, which may influence the results of this type of research. Unfortunately, there is a lack of this vital research among Asian populations. Therefore, the present study uses Han Chinese males as research subjects and focuses on the C → G mutation of the gene's no. 3175 nucleotide (apoCIII C3175G) and the T → G mutation of the no. 3206 nucleotide (apoCIIIT3206G) to explore the relationships between their variations and serum lipid levels.

## 2 Subjects and method

### 2.1 Research subjects

The Han Chinese nationality is the largest ethnic group in the world, accounting for one-fifth of the world's population, over 90% of the population of China, 98% of the population of Taiwan, 74% of the population of Singapore and 24.5% of the population of Malaysia (Wen et al., 2004). Since the formatted genotype of the Han nationality is stable due to thousands of years of genetic evolution, it is indeed a representative sample for Chinese even the Asian groups. We chose two district hospitals in Chongqing—with relatively large floating populations to cover as many genotypic Chinese backgrounds as possible, so that the surveyed sample would have high validity and reliability. To ensure an adequate number of participants, the sample size was calculated by a statistical formula. Both two hospitals are responsible for the regular check-up of the workers. Ultimately, 337 healthy male physical examination patients were randomly selected from two hospitals. All subjects were individuals of the Han nationality.

### 2.2 Method

#### 2.2.1 Clinical detection

Height, weight, waist circumference, hip circumference, blood pressure, and biochemical serum lipid indexes of all study subjects were measured. The physique index (BMI) = body weight (kg) / height^2^ (m^2^) and the waist to hip ratio (WHR) = waist circumference / hip circumference were calculated. People who have insulin resistance (IR) should be identified since IR has proved to be associated with lipids (Samuel et al., 2010).

After that, there was a questionnaire being interviewed by the participated group, which is about the factors that may affect the lipid parameters including occupation, cigarettes, physical activity, alcohol intake and diet (Xie et al., 2013).

#### 2.2.2 Specimen collection and treatment

The stomachs of the subjects were kept empty for 12 h and then 5 ml of peripheral venous blood was drawn. From this amount, 2 ml was added to a dry tube for the measurement of serum lipids and 3 ml was added to a tube containing the anticoagulant EDTA (final concentration = 0.2 g/l); this tube was inverted several times to thoroughly mix the blood sample with the anticoagulant. DNA was extracted from this sample using the QIAGEN blood DNA extraction kit and then stored at 4 °C.

#### 2.2.3 Lipid panel screen

Total cholesterol (TC), triacylglycerol (TG), high density lipoprotein-cholesterol (HDL-C) and low density lipoprotein-cholesterol (LDL-C) were all detected by an adapted oxidase method using an automatic biochemical analyser (Beckman Lx20, Beckman Coulter, Brea, California).

#### 2.2.4 ApoCIII gene amplification and DNA sequencing

Primer Premier 5.0 software was used to carry out the primer design on the ApoCIII gene. The upstream primer: 5′ TAG GGG CTG GGT GAC CGA TG 3′ (20 bp); the downstream primer: 5′ CTT GCG ACG GCC CAC TCA TAG 3′ (21 bp). The amplified fragment of 327 bp included ApoC3 C3175G (Sacl's GATC −/+) and ApoC3 T3206G. The primers were synthesised by the Shanghai Sangon Biological Engineering Technology & Service Co., Ltd.

PCR system (20 μl): 50 μmol/l upper primer 1 μl, 50 μmol/l down primer 1 μl, 10 × buffer 2 μl, 2.5 mmol/l MgCl_2_ 2 μl, 10 mmol/l dNTP 1 μl, 5 U/μl Taq enzyme 0.4 μl, H_2_O 9.6 μl, DNA 3 μl.

PCR reaction conditions: 96 °C for 5 min (94 °C for 50 s, 68 °C for 50 s) recycles 34 times, 72 °C for 10 min.

The product of the ApoC3 PCR was sent to Shanghai Sangon Biological Engineering Technology & Service Co., Ltd. Sequencing primer: 5′ CTT GCG ACG GCC CAC TCA TAG 3′.

#### 2.2.5 Statistical analysis

After collecting the data and inputting it to Excel 2003, it was analysed by SAS 9.03, followed by the application of descriptive statistical analysis. Mean, median and standard deviation (SD) were used to summarize the numerical variables, frequencies and percentages for the categorical variables. Cross-tabulations were performed with chi-square or Fisher's exact test to compare allele frequency. Analysis of variance (ANOVA) was used to compare the different serum lipid levels. The variables with P < 0.05 associated with TG were input to a stepwise multiple unconditional logistic regression for predicting the outcome based on one or more predictor variables. A P value of < 0.05 was considered significant in the model.

#### 2.2.6 Ethical considerations

Ethical approval was obtained from the managements of the two survey hospitals. Participants agreed to become involved in this research based on the principles of equality and voluntariness. The collected data were analysed only for the purposes of this research. Other than the members of the experimental group, no other individual, group or institution had access to this data.

## 3 Results

### 3.1 Basic characters

A total of 337 men were investigated in this survey. The mean age of the participants was 48.65 ± 4.04 years; the average BMI is 25.38 ± 3.17 kg/m^2^. Demographic characteristics are shown in Table 1.

**Table 1:** Characteristics of the surveyed men.

Variable	n	Mean	Std dev	Minimum	Maximum
AGE (year)	337	48.65	4.04	39.00	68.00
WHR (m)	337	0.93	0.05	0.79	1.10
BMI (kg/m^2^)	337	25.38	3.17	17.48	34.33
SBP (mm Hg)	337	124.04	14.31	81.00	179.00
DBP (mm Hg)	337	80.03	12.56	53.00	120.00
Weight (kg)	337	72.60	9.74	53.00	104.00

### 3.2 The polymorphism of apolipoprotein CIII

**Fig. 1:**
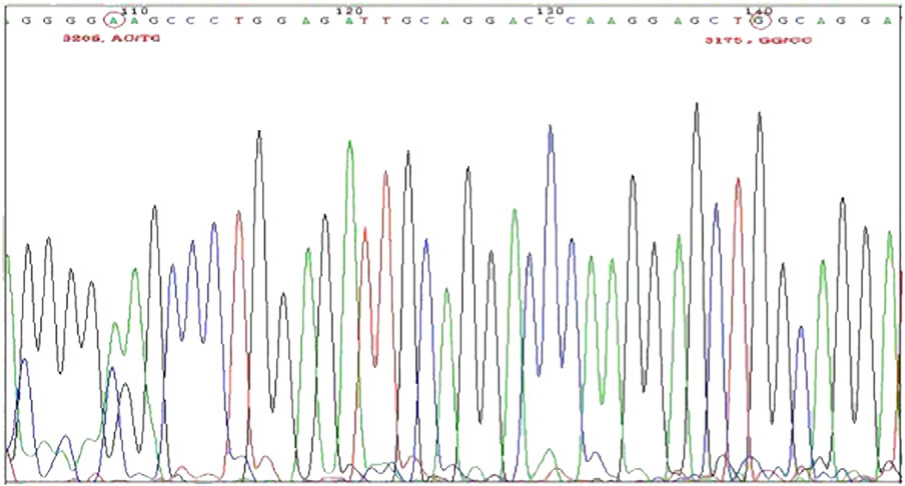
Results of the apoCIII DNA sequencing product (C3175G and T3206G) sample.

The amplified fragment of the polymorphism of apoCIII was 327 bp. The DNA sequencing result (Fig. 1) shows that the apoCIIIC3175G gene had 3 genotypes: CC type, CG type and GG type. The genotype frequencies of CC, CG and GG were 51.93%, 40.06% and 8.01%, respectively. The allele frequencies of C and G were 71.96% and 28.04%, respectively. After inspection, the polymorphism genotype distribution of apoCIIIC3175G followed the Hardy–Weinberg equilibrium principle (*x*^2^ = 4.168, P > 0.01), which showed that the selected specimen was representative of the population. The apoCIIIT3206G gene had 3 genotype results: TT type, TG type and GG type (Fig. 1). The genotype frequencies of TT, TG and GG were 6.82%, 32.34% and 60.83%, respectively. The allele frequencies of T and G were 23.0% and 77.0%, respectively. After inspection, the polymorphism genotype distribution of T3206G followed the Hardy–Weinberg equilibrium principle (*x*^2^ = 2.078, P > 0.05).

### 3.3 The relationship between the apoCIII gene polymorphism and serum lipids

According to the polymorphism genotype grouping of apoCIIIC3175G, ANOVA was carried out through pairwise comparison among the 3 groups (CC type, CG type and GG type). The results show that the GG genotype had a higher TG level than the CG genotype and the CC genotype (P < 0.05). There were no significant differences in the compared results in the levels of TC, HDL-C and LDL-C. The results are indicated in Table 2.

**Table 2:** The relationship between apoCIII C3157G/T3206G gene polymorphisms and blood lipid levels $\left(\bar{x}\pm s\right)$.

Variable	ApoCIII^3175^			ApoCIII^3206^		
	CC (n = 175)	CG (n = 135)	GG (n = 27)	TT (n = 23)	TG (n = 109)	GG (n = 205)
Age	49.12 ± 3.95	48.22 ± 4.07	47.25 ± 3.80	49.48 ± 4.16	48.72 ± 3.87	48.47 ± 4.09
TC (mmol/l)	5.23 ± 0.97	5.25 ± 0.85	5.40 ± 0.65	5.46 ± 1.24	5.21 ± 0.98	5.24 ± 0.81
TG (mmol/l)	1.87 ± 1.24	2.05 ± 1.45^⁎^	2.54 ± 1.97^⁎,△^	1.65 ± 0.87	2.08 ± 1.41	1.99 ± 1.45
HDL-C (mmol/l)	1.18 ± 0.42	1.15 ± 0.42	1.09 ± 0.22	1.22 ± 0.32	1.19 ± 0.51	1.14 ± 0.35
LDL-C (mmol/l)	3.21 ± 0.82	3.17 ± 0.79	3.14 ± 0.63	3.46 ± 1.01	3.16 ± 0.81	3.18 ± 0.76
WHR	0.94 ± 0.04	0.93 ± 0.05	0.92 ± 0.05	0.94 ± 0.04	0.93 ± 0.05	0.94 ± 0.05
BMI	26.46 ± 5.50	25.73 ± 3.66	25.04 ± 2.43	25.52 ± 3.50	25.16 ± 2.94	26.59 ± 4.43

*: P < 0.05, GG vs. CC; △: P < 0.05, GG vs. CG.

Data from the American Heart Association, the normal value of TG of human is: < 1.70 mmol/l. The critical value of clinical treatment is 2.26 mmol/l (American Heart Association, nd). In the present research, in order to explore whether different genotypes are significant in hypertriglyceridaemia (HTG) patients, the surveyed population was layered according to the TG critical value (TG < 2.26 mmol/l and TG ≥ 2.26 mmol/l).

There were 239 cases with TG < 2.26 mmol/l and 98 cases with high TG hyperlipidaemia (TG ≥ 2.26 mmol/l) in the population, and there were no significant differences in lipid groups and BMI and WHR when compared according to genotype. After analysing the relationship between genotype and various serum lipid parameters in the two subgroups, the results show that G gene loci of apoCIII^3175^ can significantly increase TC and LDL-C while G gene loci of apoCIII^3206^ can decrease it (P < 0.05). These results are indicated in Table 3.

**Table 3:** The relationship between apoCIII C3157G/T3206G gene polymorphisms and high TG levels $\left(\bar{x}\pm s\right)$.

Variable	ApoCIII^3175^			ApoCIII^3206^		
	CC (n = 49)	CG (n = 36)	GG (n = 13)	TT (n = 8)	TG (n = 36)	GG (n = 54)
TC (mmol/l)	5.12 ± 0.52	5.24 ± 0.78	5.78 ± 0.67^⁎,△^	6.69 ± 1.06	5.64 ± 0.95	5.29 ± 0.71^⁎,△^
HDL-C (mmol/l)	1.11 ± 0.52	0.98 ± 0.14	0.96 ± 0.16	1.14 ± 0.25	1.13 ± 0.59	0.97 ± 0.16
LDL-C (mmol/l)	2.83 ± 0.60	3.01 ± 0.52	3.44 ± 0.96^⁎,△^	4.57 ± 0.78	3.26 ± 0.83	2.99 ± 0.61^⁎,△^
WHR	0.95 ± 0.04	0.94 ± 0.03	0.93 ± 0.04	0.96 ± 0.03	0.94 ± 0.04	0.95 ± 0.04
BMI	30.73 ± 2.72	26.27 ± 2.92	25.81 ± 2.89	26.49 ± 1.90	25.49 ± 2.43	30.71 ± 2.73

*: P < 0.05, GG vs. CC/TT; △: P < 0.05, GG vs. CG/TG.

### 3.4 Multiple unconditional logistic regression analysis for triglycerides

TG was used as the dependent variable (Variable assignment: TG ≥ 2.26 = 1; TG < 2.26 = 0); all the independent variables: Smoking (Yes = 1; No = 0), Physical Activity (No = 1; Yes = 0), alcohol intake (Yes = 1; No = 0), insulin residence (Yes = 1; No = 0), age (age ≥ 50 = 1; age < 50 = 0), BMI (BMI ≥ 25 = 1, BMI < 25 = 0), diet (No = 1; Yes = 0), WHR (WHR ≥ 0.9 = 1, < 0.9 = 0), occupation (highly consideration work = 1, common work = 0), apoCIII^3175^ and apoCIII^3206^ were included in a stepwise logistic regression and then variable Age, BMI, WHR, apoCIII^3175^ and apoCIII^3206^ remained into the final logistic regression model. After the last screening, the results show that apoCIII^3175^ and apoCIII^3206^ gene are significant variables. Compared with apoCIII^3175^ CC gene carriers, CG and GG gene carriers are almost two times more likely to increase TG (P < 0.01). Compared with apoCIII^3206^ TT gene carriers, TG gene carriers are 0.34 times less likely to increase TG (P < 0.01, Table 4).

**Table 4:** Multiple unconditional logistic regression analysis for TG.

Gene	Genotype	Adjusted OR (95% CI)	Adjusted P-value
ApoCIII^3175^	CC	1.00	
	CG	1.991 (1.475–2.687)	< 0.01
	GG	1.967 (1.406–2.752)	< 0.01
ApoCIII^3206^	TT	1.00	
	TG	0.336 (0.219–0.516)	< 0.01
	GG	1.358 (0.953–1.935)	0.0904

Multiple unconditional logistic regression, adjusted by AGE, BMI, and WHR.

## 4 Discussion

In summary, this research shows that the polymorphism loci of apoCIIIC3175G are closely related to the levels of TG in Han Chinese males. The plasma triglyceride level of the homozygous (GG genotype) carriers is higher than that of CG genotype carriers while the plasma triglyceride level of the CG genotype is higher than that of the CC genotypes. However, this research did not find a correlation between various genotypes of apoCIIIT3206G and TG, which is consistent with the findings of Herbeth et al. (2007). For this result, G gene loci of apoCIII^3175^ may have influenced the synthesis of TG lipase, but the conclusion still needs further study.

After further classification of TG (TG < 2.26 mmol/l group and TG ≥ 2.26 mmol/l group), the results show that apoCIII gene is associated with TG and LDL-C in the relatively high TG group, and that the G allele of apoCIII^3175^ has a tendency to increase cholesterol and low density lipoprotein levels but G allele of apoCIII^3206^ has an inverse effect on TC and LDL-C. Nevertheless, there were no significant differences in the normal TG group. This means that the mutation of the gene is more closely related to serum lipid levels in high TG group than the normal TG group. Thus, if an HTG patient carries the apoCIII^3175^ GG genotype, his lipid levels should be closely monitored, since he is a more likely candidate for cardiovascular disease than the other genotypes. On another hand, the mechanism of G gene loci of apoCIII^3206^ for decreasing TC and LDL-C in HTG should be explored, so it can be applied as a preventive measure in the future.

There are two possible reasons that the results comparing genotypes are not the same in the entire participant group and HTG groups. On one hand, the researched population has different heterogeneities. Therefore, the various genetic backgrounds may have influenced the relationships between serum lipid levels and apocIII (Li et al., 2010). On the other hand, the level of serum lipids is affected by environmental factors, such as the effects of dietary habits and lifestyle. Therefore, the actions between lipids may strengthen or weaken the actions on gene loci. But when it is divided by TG content, the TG hybrid factors action is under control, so the correlation between apoCIII gene and serum lipid can be presented.

The results of TG multiple unconditional logistic regression in this research showed that TG level is significantly influenced by the G gene loci of apoCIII^3175^ and apoCIII^3206^, and that result was adjusted by the age, WHR and BMI. This conclusion is consistent with the results in the HTG group which shows that people carrying G gene loci of apoCIII^3175^ are more likely to increase TG than C gene loci carriers. In contrast, people carrying G gene loci of apoCIII3206 are more likely to decrease TG than T gene loci carriers. In future research, it would be beneficial to determine the gene constitution that indicates an individual is liable to develop TG; this finding would allow preventive intervention for those at risk.

This study aimed to fill the gap in the research regarding the relationship between apoCIII polymorphism and serum lipids in the Asian population. The method adopted advanced equipment to ensure that the results were reliable. Nevertheless, this type of study still needs to be conducted on larger samples from a wider range of districts. Hence, we plan to conduct a multicentre experiment with a scaled up sample range to further confirm the results.

## 5 Conclusion

This research shows that the G gene loci of apoCIIIC3175G polymorphism are a dangerous factor for increases in individuals' triglyceride levels. Therefore, determining the high-risk indicators is an efficient estimation of serum lipid content and is an economical method for presumptive diagnosis and recognizing that treatment for HTC is required. On the other hand, G gene loci of apoCIIIT3206G are beneficial for decreasing TG in the common group and TC and LDL-C in the HTG group. These findings should be further studied in order to adopt new method to prevent and treat hyperlipidaemia.
